# Non-*aureus* Staphylococci Cause the Spontaneous Cure or Persistent Infection of Major Bovine Mastitis Pathogens in the Murine Mammary Glands

**DOI:** 10.3390/ani14233526

**Published:** 2024-12-06

**Authors:** Witaya Suriyasathaporn, Aphisek Kongkaew, Montira Intanon, Anyaphat Srithanasuwan, Duanghathai Saipinta, Noppason Pangprasit, Atigan Thongtharb, Areerat Chuasakhonwilai, Wasana Chaisri

**Affiliations:** 1 School of Veterinary Medicine, Faculty of Veterinary Medicine, Chiang Mai University, Chiang Mai 50100, Thailand; witaya.s@cmu.ac.th (W.S.); montira.intanon@cmu.ac.th (M.I.); anyaphat.sw@gmail.com (A.S.); duanghathai.s@cmu.ac.th (D.S.); atigan.t@cmu.ac.th (A.T.); 2 Research Center of Producing and Development of Products and Innovations for Animal Health and Production, Chiang Mai University, Chiang Mai 50100, Thailand; 3 Cambodia Campus, Asian Satellite Campuses Institute, Nagoya Universities, Nagoya 464-8601, Japan; 4 Research Administration Section, Faculty of Medicine, Chiang Mai University, Chiang Mai 50200, Thailand; aphisek.k@cmu.ac.th; 5 Department of Animal Sciences, Wageningen University, 6708 PB Wageningen, The Netherlands; 6 Akkhraratchakumari Veterinary Colleges, Walailak University, Nakhon Si Thammarat 80161, Thailand; p.noppason@gmail.com; 7 Office of Research Administration, Chiang Mai University, Chiang Mai 50200, Thailand; areeratc.yok@gmail.com

**Keywords:** mastitis, mouse mastitis model, non-aureus staphylococci, major mastitis pathogen, intramammary infection

## Abstract

The microbial ecology in mastitis involves the interactions between bacteria and the mammary gland environment. Poor mastitis control, for which understanding these microbial relationships is crucial, increases the risk of mastitis and co-infections. The aim of this study was to determine the pathogenesis and bacterial ecology of murine mammary glands following intramammary infection (IMI) with *S. aureus* (AU), *S. agalactiae* (SA), and four isolates of selected non-aureus staphylococci (NAS), as well as co-infections of AU or SA with NAS. Selected NAS demonstrated resistance to growth inhibition after co-culture with AU and SA and were proven to be protective in vitro against AU and SA. Both single infections and co-infections of AU, SA, two selected isolates of *S. hominis* (NAS1 and NAS2), and two selected isolates of *S. chromogens* (NAS3 and NAS4) were injected into the murine mammary glands at 10^5^ CFU/mL. After 72 h of inoculation, the results showed that AU, AU–NAS2, and AU–NAS3 expressed severe inflammation with clinical signs of mastitis. The persistence of AU was found for AU, NAS3, and AU–NAS3, whereas the persistence of SA was found for SA–NAS1, SA–NAS3, and SA–NAS, although single SA could not persist. Interestingly, NAS2 in both the single- and co-IMIs with AU or SA resulted in a spontaneous cure 72 h after the IMI. In conclusion, some NAS have beneficial results because they can eliminate *S. aureus* and *S. agalactiae*, but some NAS have negative effects because they can support persistent *S. agalactiae* infection. These results may be used to explain both the advantages and disadvantages of NAS on farms with poor mastitis control. In addition, the beneficial *S. hominis* may initiate natural live antibiotics and reduce antibiotic resistance problems.

## Simple Summary

The aim of this study was to determine the possible interaction of experimental intramammary co-infection in a murine mastitis model. A single *S. aureus* (AU) and *S. agalactiae* (SA); selected non-aureus staphylococci (NAS), such as *S. hominis* and *S. chromogenes*; and a mix of these were experimentally injected into mouse mammary glands. Selected NAS that previously demonstrated resistance to growth inhibition after co-culture with AU and SA were used. The selected isolates of *S. hominis* after co-infection with AU or SA resulted in spontaneous cures. In contrast, some *S. hominis* and *S. chromogenes* supported the persistence of SA after co-infection.

## 1. Introduction

Mastitis is an interaction of bacteria and the mammary gland environment of dairy cattle. Bovine milk may contain a wide range of microbial species, including both beneficial and harmful bacteria. Co-infection occurs when multiple bacterial species simultaneously infect the mammary gland. These bacteria coexist in a variety of ways, with some benefiting or cooperating with others, whereas some compete with one another [1]. After infection, this forms a miniature ecosystem, where the development of innate or adaptive immunity to destroy invading bacteria results in a spontaneous cure after an acute infection [2,3]. However, a subset of phylogenetically diverse bacteria can establish persistent infection and modify the intracellular environment within hosts [2,3]. A persistent infection can also be modified with subsequent reinfection and co-infections, as shown in many diseases, such as human bacterial vaginosis [4] and bovine mastitis [5,6]. The ecological interactions between the co-infection’s bacteria and the host are crucial for bacterial survival or death in polymicrobial infection communities [5,6]. Our previous in vitro study [5] suggested that specific strains of *S. chromogenes* and *S. hominis* play a role in the natural spontaneous curing of mastitis from *S. aureus* and *S. agalactiae*, which are both major and persistent pathogens through either destroying the infection or surviving in the udder to accelerate the udder’s defense mechanism. An experimentally infected model is needed to enhance the possibility of those specific bacteria with bacteriolytic capabilities for possible novel mastitis treatment.

As contagious and persistent mastitis pathogens, *Staphylococcus aureus* [7,8] and *Streptococcus agalactiae* [9] are the leading causes of bovine mastitis worldwide. In recent decades, the common non-pathogenic coagulase-negative staphylococci (CNS) or non-aureus staphylococci (NAS) have been discovered to bring about a short-term spontaneous cure [10]. Still, the remaining chronic subclinical mastitis has become the most common type in bovines [11,12]. The NAS species originate from distinct habitats and show clear differences that may be related to their diverse ecology and epidemiological behavior [13]. Regarding co-infection, the effects of NAS on *Staphylococcus aureus* intramammary infection (IMI) risk have yielded an ongoing debate [14]. In 2018, the protective effect of NAS, through a reduction in the virulence factor expression of *S. aureus*, was found in some species [15]. Priming the murine mammary gland with *S. chromogenes* IM suppressed *S. uberis* growth [16]. A recent study found that most NAS declined after co-culture with *S. aureus* and *S. agalactiae* [5]. Nevertheless, very few isolates of NAS, with *S. hominis* and *S. chromogenes* as examples, resist *S. aureus* and *S. agalactiae* [5]. Some NAS strains, such as *S. chromogenes*, synthesize and secrete 6-thioguanine (6-TG), a molecule found to inhibit agr quorum sensing, resulting in a reduction in the virulence and growth of *S. aureus* [17]. The secretion of antibacterial peptides, such as bacteriocins, by NAS species is a potential method for inhibiting the primary mastitis pathogen [18]. In addition, NAS’s ability to compete with major mastitis pathogens may be influenced by a variety of ecological features, including the production of enzymes, siderophores, and biofilm formation. These kinds of isolates might play a role in the natural spontaneous curing of mastitis from *S. aureus* and *S. agalactiae* by either destroying the infection or surviving in the udder to accelerate its defense mechanism. This indicates that the concept of novel bacteria with bacteriolytic capabilities might lead to new mastitis treatments.

To continue to understand the protective effects of NAS in the co-infection ecology with *S. aureus* and *S. agalactiae*, NAS isolates proven to be protective were used to determine the beneficial effects after being co-infected with *S. aureus* and *S. agalactiae*. The selected isolates had resisted the growth of *S. aureus* and *S. agalactiae* after in vitro co-culture in our previous study [5]. Therefore, the present study aimed to determine the persistence of mastitis pathogens and the protective effect of selected NAS against *S. aureus* (AU) and *S. agalactiae* (SA) in mouse mammary glands, as well as a histopathological analysis after experimental single- and co-IMIs of NAS with AU and SA. When pathogens infect a host, the resident bacteria play a critical and often protective role during infections, both by modulating the immune system responses and mediating colonization resistance [19]. Therefore, the microbial shift was also investigated following the experimental IMIs.

## 2. Materials and Methods

Based on our previous study [5], we selected *S. aureus* ATCC25923 (AU), a field isolate of *S. agalactiae* (SA), and four NAS isolates proven to be protective, including two isolates of *S. hominis* (NAS1 and NAS2) and two isolates of *S. chromogenes* (NAS3 and NAS4). These isolates were proven to be protective by means of resisting the growth of *S. aureus* and *S. agalactiae* after in vitro co-culture. All the NAS isolates were originally collected from subclinical mastitis cows and then tested for their survival and resistance performances after co-culture with *S. aureus* or *S. agalactiae* [5]. The best-performing NAS isolates were selected. After recovery, the inoculums were cultured on Tryptic Soy Agar (Merck, Darmstadt, Germany) and incubated at 37 °C for 18–20 h. The bacterial colonies were suspended in sterile PBS, adjusted to 10^8^ CFU/mL, and then diluted in sterile PBS to reach the desired concentration of 10^5^ CFU/mL. The bacterial inoculum size was determined by serially diluting the culture and drop-plating the dilutions onto plate count agar (Merck, Darmstadt, Germany).

All the mice were purchased from the National Laboratory Animal Center, Mahidol University, and transported to the Laboratory Animal Center for study. All the procedures involving animals were approved by the committee of the Laboratory Animal Center, Chiang Mai University (MC009/2566 [03/2566-09-19]). The animals’ care and treatment were carried out in accordance with the institutional guidelines. Fifteen lactating CD-1 mice, 10–12 days post-birth, were randomly assigned to experimental inoculation with one of six single bacteria (AU, SA, NAS1, NAS2, NAS3, and NAS4), eight co-infected bacteria (AU–NAS1, AU–NAS2, AU–NAS3, AU–NAS4, SA–NAS1, SA–NAS2, SA–NAS3, and SA–NAS4), or PBS as a control. The IMIs were performed as described by Camperio et al. [20], with some modifications. Two hours prior to inoculation, the pups were separated. Then, 100 µL of an overnight culture of all the NAS, *S. aureus*, and *S. agalactiae* (approximately 10^5^ CFU/mL) were inoculated individually and co-injected into both sides of the inguinal mammary gland of each mouse using syringes with 33-gauge needles.

Seventy-two hours post-infection, all the mice were euthanized by cervical dislocation. Both of the injected inguinal mammary glands were aseptically collected, weighed, and homogenized in 2 mL of sterile phosphate buffer saline (PBS). They were then serially diluted (1:10). Fifty microliters of dilution was plated onto mannitol salt agar (for *S. aureus*/Staphylococci) and Edwards agar (for *S. agalactiae*) and incubated at 37 °C for 24–48 h. Each dilution was performed in duplicate. The bacterial colonies ranged from 25 to 250 and were calculated and expressed as colony-forming units per gram (CFU/g) of the mammary gland. All the colonies were picked and confirmed for bacterial identification using MALDI-TOF mass spectrometry (Bruker Daltonik GmbH, Bremen, Germany). With respect to the score analysis, a secure genus identification or probable species identification was determined with a MALDI Biotyper score range of 2.000–2.299, with a highly probable species identification scoring 2.300–3.000. The main spectrum in this protocol was obtained using the MALDI Biotyper automated FlexControl software, version 3.0 (Bruker Daltonik GmbH, Bremen, Germany).

**Figure 1:**
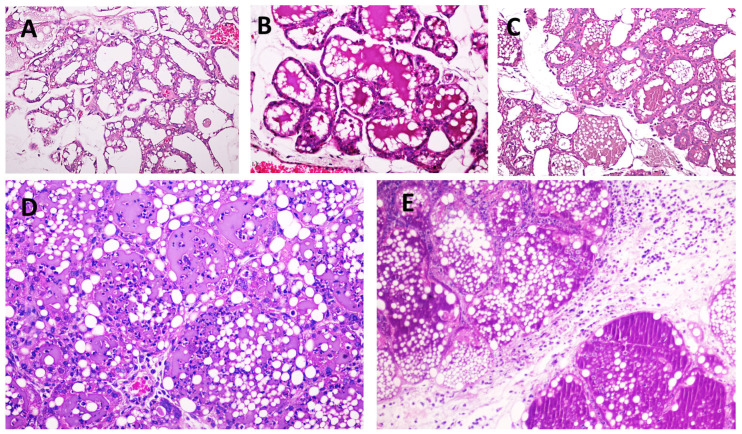
The histopathological scores are based on the histological changes in the murine mammary glands, with some modifications. (**A**): healthy, score 0, no infiltration, and undamaged tissue; (**B**): mild, score 1, and polymorphonuclear cell (PMN) infiltration in isolated areas of the tissue sections; (**C**): moderate, score 2, and PMN infiltration in the alveolar and interstitial areas; (**D**): severe, score 3, PMN infiltration, and focal areas of tissue damage; and (**E**): very severe, score 4, heavy PMN infiltration, and extensive necrotic areas.

Before euthanasia, the mouse mammary glands were observed for clinical signs, including heat, pain, redness, and swelling, and were then categorized as with or without clinical signs. The mammary gland samples from all the groups were fixed in 10% neutral buffered formalin, followed by routine processing to obtain 4 μm paraffin-embedded histological sections. These sections were then stained with hematoxylin and eosin and examined using light microscopy. The histological findings were categorized according to Camperio et al. [20] with modifications, assessing the amounts of infiltrated inflammatory cells and the levels of tissue damage and necrotic tissue in the alveolar area. The categorizations were as follows: score 0 (healthy), no infiltration and undamaged tissue; score 1 (mild), polymorphonuclear cell (PMN) infiltration in isolated areas of the tissue sections; score 2 (moderate), PMN infiltration in the alveolar and interstitial areas; score 3 (severe), PMN infiltration and focal areas of tissue damage; and score 4 (very severe), heavy PMN infiltration and extensive necrotic areas, as shown in Figure 1.

Considering the results from 72 h after the IMIs, complex symbiotic relationships are proposed based on the persistence of the bacteria of major pathogens (AU and SA) and NAS isolates (NAS1–NAS4) in the animal host’s udder ecosystem, which is composed of leukocytes and mammary gland cells. The relationships in the infections in this study were categorized into “persistence”, when major pathogens, NAS isolates, or both were found, and “spontaneous cure”, when AU, SA, and NAS1–NAS4 were absent.

## 3. Results

Table 1 shows the results of the IMIs after 72 h. The control group had no clinical signs and had *S. lentus* as the resident microbes with fewer than 10 CFU/mL. For the infection groups, most of the resident microbes found were *S. lentus* and *S. xylosus*. All the mice without clinical signs, with no existing IMI species, and with histopathological scores up to 2 were consistently found to have a small amount of resident microbes at <10 CFU/mL. The mammary glands infected with AU, including AU, AU–NAS2, and AU–NAS4, exhibited clinical signs, including swelling, hardness, and redness.

For the single IMIs, the microbiological results revealed that SA and both isolates of *S. hominis* (NAS1 and NAS2) could not survive in the mammary gland 72 h post-infection. In contrast, AU and both isolates of *S. chromogenes* (NAS3 and NAS4) were able to survive in the mammary gland. The number of bacteria remained high in AU and NAS3 but low in NAS4. No resident microbes were found in the IMI udder with AU.

With respect to the co-IMIs, the numbers of the existing microorganisms at 72 h varied from 0 for AU–NAS2 to >10^5^ CFU/mL for AU–NAS3. The co-IMIs with both NAS2, including AU–NAS2 and SA–NAS2, and AU–NAS1 found no persistent IMI bacteria. For the other groups, all the NAS also failed to survive at 72 h after the IMI when cultured with either AU or SA. In comparison to the single-AU IMI, AU could persist in the mouse mammary gland only when AU was co-infected with NAS3 and NAS4.

**Figure 2:**
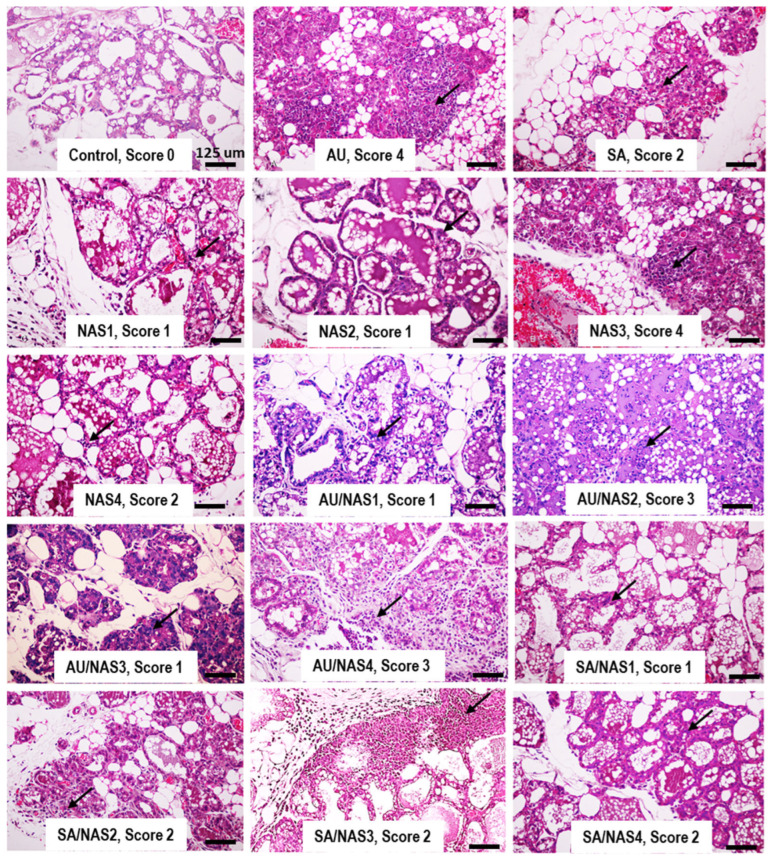
Pathohistological features and scores of the murine mammary glands at 72 h after the experimental IMIs: either the single IMIs with *S. aureus* (AU), *S. agalactiae* (SA), *S. hominis* (NAS1 and NAS2), and *S. chromogenes* (NAS3 and NAS4), or the mixed IMIs with AU and SA together with NAS1–NAS4. The details of the scores are described in Figure 2. The arrows indicate the leukocyte influx.

The histopathological scores of all the groups are shown in Table 1. Figure 2 provides examples of the histological characteristics of the murine mammary glands at 72 h after the single or dual IMIs with AU, SA, and NAS. The control, an intramammary inoculation with PBS, showed no inflammatory signs in its histopathological appearance (Figure 2). With a high number of existing microbes at 10^4^ CFU/mL 72 h after the IMI, the single IMIs with AU and NAS3 resulted in pathohistological scores of 4 (Table 1), indicated by very severe inflammation and characterized by extensive neutrophil infiltration and a necrotic area on the ducts and alveoli (Figure 2). The mammary glands with clinical signs, including AU, AU–NAS2, and AU–NAS4, had scores of 4, 3, and 3, respectively. The pathohistological scores for the single or dual IMIs with SA were 1 for SA–NAS1 and SA–NAS4 and 2 for SA, SA–NAS2, and SA–NAS3.

## 4. Discussion

This study used a murine mastitis model to understand the results of the IMIs in various circumstances: after a single infection with contagious pathogens, such as *S. aureus* and *S. agalactiae*; or with selected protective-proven NAS; or the co-infection of these pathogens that occur in dairy cattle. An intraductal mouse model has been proven to mimic bovine CNS mastitis and has the potential as a complementary in vivo tool for future application in CNS mastitis research [21]. Therefore, the selected NAS isolates might not be representative of other NAS that interact with AU and SA in different ways [5].

The results of *S. agalactiae* (SA) and *S. hominis* (NAS1 and NAS2) in the single infection were spontaneous cures, and moderate and mild histopathological scores without any clinical signs were found (Figure 2). The mild score from *S. agalactiae*-infected mouse mammary glands with spontaneous cures in this study was in accordance with a few cases of short spontaneous cures of natural *S. agalactiae* bovine IMIs in our previous longitudinal study [22]. In a mouse model, a single *S. agalactiae* IMI showed a pronounced tendency towards spontaneous clearance by exhibiting both innate and acquired immune responses [23]. However, most or 88% of the natural bovine IMIs with *S. agalactiae* were not spontaneous cures, indicating persistent infection in this pathogen [22]. Globally, bovine mastitis infected by *S. agalactiae*, a contagious and rarely spontaneous resolve, is still prevalent in many dairy farms worldwide [24]. Our result in this study also supports the most persistent SA after the co-IMIs of SA with either NAS1, NAS3, or NAS4 (Table 1). Outbreaks of *S. agalactiae* mastitis have been reported to be associated with poor mastitis control in many countries, for example, China [24], Thailand [22,25], and Vietnam [26]. Regardless of the pathogens, the mastitis control program, relating to improved sanitation such as enhanced milking hygiene, implementation of post-milking teat disinfection, and maintenance of milking machines, is a general measure to prevent new cases of mastitis. Our finding might be the reason for the outbreak of *S. agalactiae*, which always occurs in a herd with a poor mastitis control program. Another explanation for our finding might be the use of our specific field strain of SA in this study. Numerous studies have demonstrated the genomic diversity of *S. agalactiae* [24,25,27]. In general, most strains of *S. agalactiae* had low spontaneous cures and were always persistent pathogens, but very few isolates had spontaneous cures [28]. The variation in this transmission dynamic might be due to the expression of the virulence characteristics, including efficient growth in milk, elevated biofilm formation ability, and strong adhesion ability, causing the high prevalence of the specific *S. agalactiae* strains in the bovine environment [24]. However, the mechanism of persistent *S. agalactiae* after co-infection with NAS needs to be confirmed, and further investigations should be conducted.

*S. hominis* has been reported to result in a spontaneous cure after an IMI in mice. In comparison to *S. chromogenes*, a recent study showed that bovine neutrophils had more viability and a higher level of susceptibility to neutrophil killing [29]. In contrast, inoculating mice with *S. aureus* or *S. chromogenes* at a dosage of 10^4^ CFU/udder induced clinical signs and a resultant histopathological score of 4 with persistent bacteria. Krishnamoorthy et al. [30] reported that mice infected with NAS species displayed fewer histopathological alterations compared to those infected with *S. aureus*, which is consistent with our findings.

The co-IMIs in this study were the first such experimental IMIs in mice. Both persistent and spontaneous cures were found (Table 1). NAS2 resulted in the absence of both *S. aureus* and *S. agalactiae* within the host, while NAS1 only led to the absence of *S. aureus*. The inhibitory effects of NAS might directly inhibit the growth or activity of other bacteria through mechanisms like producing antimicrobial substances [31] or triggering immune responses that affect both bacteria negatively. The possibility of an absence of *S. aureus* or *S. agalactiae* after co-infection with a certain isolate of NAS could be explained by an increase in the influx of white blood cells, resulting in a natural spontaneous cure [6]. In addition, despite their prevalent commensal nature, certain isolates of NAS have been observed to exert inhibitory effects on the proliferation or survival of pathogenic species in the same environment [32]. Similarly, Srithanasuwan et al. [5] and De Vliegher et al. [33] reported that NAS, particularly some strains of *S. chromogenes,* can inhibit the growth of major mastitis pathogens, such as *S. aureus*, *S. dysgalactiae*, and *S. uberis*, in vitro. Regarding the bacterial ecology, none of the NAS isolates were found at 72 h after the co-IMIs with AU and SA. In competition with AU in vitro, all the NAS, except *S. Hominis* (NAS2), showed fewer colonies when cultured in the same medium [5]. The inhibitory effect observed for *S. agalactiae* against all the NAS might be due to agalacticin and the antibiotic nisin P produced by *S. agalactiae*, as reported by Garcia-Gutierrez et al. [34] and Amaral et al. [35]. The clinical signs and histopathological score of 3 for AU–NAS2 might be due to this specific *S. hominis* isolate stimulating a higher immune response in vitro, especially in the gene expressions of IL-1b, TNF, NOX1, TLR1, and TLR2 [29], and the mixed IMIs resulted in a higher immune response [36].

With respect to the persistence of AU or SA in the co-IMIs group, AU–NAS3, SA–NAS1, SA–NAS3, and SA–NAS4 supported the persistent subclinical mastitis of both *S. aureus* and *S. agalactiae* [35,37]. No clinical signs were reported in the mice infected with a single infection with all the NAS, even with histopathological scores of 4 for NAS3. In a study on buffalo mastitis, high histopathological scores were found in the mammary tissues without any clinical signs [38]. Interestingly, both the single-AU and single-NAS3 infections resulted in high histopathological scores of 4, but AU–NAS3 had a low histopathological score, indicating that both pathogens might inhibit the severity of each other. *S. chromogenes* releases a purine analog that inhibits *S. aureus* virulence [17]. In addition, it might be that the bacteriocins produced by the NAS could inhibit *S. aureus* growth [39] or alter the host’s immune response in a way that affects both bacteria, thereby reducing the degree of inflammation. In contrast to AU, SA, which could not persist when infected alone, persists in the mammary glands of mice 72 h after IMI when co-infected with NAS1, NAS3, and NAS4 (Table 1). This result differed from the co-IMIs of *S. chromogenes* and *S. uberis*, showing that priming the murine mammary gland with NAS reduces *S. uberis* growth [16]. Our previous study found that the order of the co-culture with NAS and the major pathogens is significantly related to survival [5].

We found resident bacteria or microbiota, such as *S. lentus*, *S. xylosus*, *S. kloosii*, and others, in the murine mammary glands (Table 1). Some of these, including *S. lentus*, have been reported previously as the most prevalent microbiota in unchallenged bovine mammary glands [21]. *S. xylosus* is a common skin commensal bacterium in humans and other mammals [40], including mice [41]. The relationships between the bacterial composition of milk and the risk of mastitis suggest that indigenous bacteria may be necessary for mammary gland health [42].

## 5. Conclusions

In this study, all the selected NAS isolates that were shown to be protective exhibited varying clearing results after co-infection with *S. aureus* and *S. agalactiae*. Both *S. hominis* isolates demonstrated beneficial outcomes because they can eliminate *S. aureus*. Still, only one strain of *S. hominis* can eliminate *S. agalactiae* when co-infected in the murine udder. In contrast, the *S. chromogenes* IMI has negative effects due to its persistence or being a carrier for *S. agalactiae* infections. Future research efforts should focus on elucidating the mechanisms underlying NAS-mediated pathogen elimination and their impact on the immune system, udder recovery time, and safety in larger animals. In addition, many properties of the strains must be demonstrated further in order to be considered for probiotic use.

## Figures and Tables

**Table 1:** Histopathological score (HS), the persistence of microorganisms, the characteristics of the udder, and the remaining resident microorganisms or microbiota after the challenge with a major pathogen or NAS and co-infection of NAS with major pathogens.

		After IMI 72 h				
IMI Species ^a^		IMI Species ^b^	CFU/mL	HS	Clinical Signs	Remaining Microbes
Control (PBS)		-	<10	0	No	*S. lentus*
Single infection						
	*S. aureus* (AU)	AU	4.0 × 10^4^	4	Yes	
	*S. agalactiae* (SA)	ng	<10	2	No	*S. xylosus*, *S. lentus*
	*S. hominis* (NAS)	ng	<10	1	No	*S. xylosus*, *C. stationis*
	*S. hominis* (NAS2)	ng	<10	1	No	*S. xylosus*
	*S. chromogenes* (NAS3)	NAS3	1.6 × 10^4^	4	No	*S. xylosus*, *S. lentus*
	*S. chromogenes* (NAS4)	NAS4	<10	2	No	*S. lentus*
Mixed infection						
	AU–NAS1	ng	<10	1	No	*S. lentus*
	AU–NAS2	ng	-	3	Yes	
	AU–NAS3	AU	>10^5^	1	No	*S. lentus*
	AU–NAS4	AU	7.9 × 10^3^	3	Yes	*S. lentus*
	SA–NAS1	SA	1.6 × 10^3^	1	No	*S. kloosii*, *S. saprophyticus*
	SA–NAS2	ng	<10	2	No	*S. kloosii*, *Cur. albidum*
	SA–NAS3	SA	6.3 × 10^2^	2	No	
	SA–NAS4	SA	1 × 10^3^	1	No	

^a^ IMI indicates the experimental intramammary infection at 0 h with *S. aureus* ATCC25923 and all other mastitis pathogens from field isolates; ^b^ “ng” indicates no growth; there is also no persistent IMI bacteria found or “spontaneous cure”, with the letters AU, SA, NAS3, and NAS4 indicating the “persistence” of the IMI bacteria.

## Data Availability

Upon reasonable request, the corresponding author can provide the datasets used and/or analyzed during the current investigation.
